# Embracing informed learner self-assessment during debriefing: the art of plus-delta

**DOI:** 10.1186/s41077-021-00173-1

**Published:** 2021-06-05

**Authors:** A. Cheng, W. Eppich, C. Epps, M. Kolbe, M. Meguerdichian, V. Grant

**Affiliations:** 1 KidSIM Simulation Program, Alberta Children’s Hospital, Departments of Pediatrics and Emergency Medicine, Cumming School of Medicine, University of Calgary, 28 Oki Drive NW, Calgary, T3B 6A8 Canada; 2 RSCI SIM Centre for Simulation Education and Research RCSI University of Medicine and Health Sciences, Dublin, Ireland; 3 Departments of Anesthesiology and Interprofessional Education, University of Tennessee Health Science Center, Memphis, USA; 4 Simulation Center, UniversitatsSpital Zurich, Ramistrasse 100, 8091 Zurich, Switzerland; 5 Department of Emergency Medicine, NYC Health + Hospitals/Harlem, NYC Health + Hospitals/Simulation Center, Columbia University, New York, USA; 6 KidSIM Simulation Program, Alberta Children’s Hospital, Departments of Pediatrics and Emergency Medicine, Cumming School of Medicine, University of Calgary, 28 Oki Drive NW, Calgary, T3B 6A8 Canada

**Keywords:** Debriefing, Plus-delta, Learner self-assessment, Feedback

## Abstract

The healthcare simulation field has no shortage of debriefing options. Some demand considerable skill which serves as a barrier to more widespread implementation. The plus-delta approach to debriefing offers the advantages of conceptual simplicity and ease of implementation. Importantly, plus-delta promotes learners’ capacity for a self-assessment, a skill vital for safe clinical practice and yet a notorious deficiency in professional practice. The plus-delta approach confers the benefits of promoting uptake of debriefing in time-limited settings by educators with both fundamental but also advanced skills, and enhancing essential capacity for critical self-assessment informed by objective performance feedback. In this paper, we describe the role of plus-delta in debriefing, provide guidance for incorporating informed learner self-assessment into debriefings, and highlight four opportunities for improving the art of the plus delta: (a) exploring the big picture vs. specific performance issues, (b) choosing between single vs. double-barreled questions, (c) unpacking positive performance, and (d) managing perception mismatches.

## Introduction

The evolution of simulation-based education in healthcare has been accompanied by growth in the number of debriefing methods, frameworks, and/or conversational strategies [1–6]. Many debriefing methods demand considerable skill, which impedes effective implementation. The plus-delta approach to debriefing has multiple benefits since it is conceptually simple and easy to implement, while promoting learner capacity for self-assessment—a skill vital for safe clinical practice [2, 5, 7–12]. With plus-delta, facilitators engage learners in a self-assessment of their own performance [12], which in turn provides opportunity for individual and team reflexivity [13, 14]. Unfortunately, many facilitators lack awareness of the importance of learner self-assessment in promoting professional practice, resulting in an inability to maximize the impact of this approach or in some cases, an avoidance of the method altogether. We believe this straightforward approach can demystify the art of debriefing and promote its uptake, while concurrently capitalizing on the benefits of informed learner self-assessment. In this paper, we clarify the implementation of plus-delta and offer strategies to best execute the approach by clearly defining the role and benefits of learner self-assessment in debriefing.

This paper has several aims, structured in a step-wise manner to guide the reader through the background, rationale, and strategies for adopting learner self-assessment in debriefing. First, we define the plus-delta approach and describe its role in debriefing. Second, we argue for the important role for incorporating informed learner self-assessment into debriefings and map debriefing strategies to Ross’ four-stage model for fostering learning through self-assessment [15]. We then describe four opportunities for fine-tuning the art of the plus-delta, namely (1) using plus-delta for the big picture vs. specific performance issues, (2) single- vs. double-barreled questioning, (3) unpacking positive performance, and (4) managing perception mismatches. To close, we discuss how to incorporate various forms of informed learner self-assessment into debriefing.

## What is plus-delta?

The plus-delta approach describes a debriefing strategy in which participants are asked to reflect on the entire simulation event (or portions thereof) and assess their individual and/or collective performance. When applying this approach, facilitators ask learners: “What went well and what would you do differently (or improve) next time?” [7, 9, 12]; “What did you do well, and what did not go well, and why?” [10]; “What was easy and what was challenging for you?” [5]; or other similar questions. Outside of healthcare, the US Army has adopted a version of this approach through a performance feedback method termed “After Action Review” [16, 17]. Following training, soldiers engage in a facilitated conversation to clarify what aspects of performance met pre-defined standards, and where there was opportunity for improvement [17]. The plus-delta approach, when coupled with feedback and teaching, can be used as the primary conversational strategy in a debriefing [7, 9–11] or used more selectively by blending it with other strategies (e.g., focused facilitation) depending on the learning context, amount of time available, and facilitator preferences (e.g., learner vs. instructor-centered debriefing) [12, 18]. Ideally, an effective plus-delta generates two lists of behaviors (i.e., things that the learners felt went well, and things that the learners felt could be improved), which then prompts further discussion, reflection, and/or learning during the debriefing. The true function of plus-delta is to conduct a learner self-assessment, the benefits and downsides of which have been extensively studied, debated, and described in the healthcare and education literature [19, 20].

## Learner self-assessment for professional development

Although traditional notions highlight the importance of self-assessment for professional development, professionals are notoriously poor at assessing their own performance [19]. In a series of educational studies, participants were recruited to self-assess themselves after performing a wide range of tasks requiring humor, logical reasoning, and English grammar. These studies found that participants in the lowest scoring quartile tended to overestimate their performance [21]. Similar patterns have been observed in healthcare providers. Physicians often fail to recognize knowledge deficits, with less experienced and/or poorer performing clinicians demonstrating a tendency to overrate their knowledge and skills [19, 22–26]. Trainees exemplify this discrepancy and consistently overestimate competency in the face of both inadequate performance and adequate performance [22–24, 26]. Even experienced clinicians sometimes struggle to accurately assess their ability to integrate skills into clinical practice [19, 25].

Despite these inaccuracies, there are several important benefits of learner self-assessment. When self-assessments are accurate, additional learning can be gained from performing the act itself, thus allowing for skill development in the absence of expert assessment [27]. Learners who engage in self-assessment set higher goals and commit more effort to the acquisition of these goals, which equates to enhanced future performance [26, 27]. Objective feedback informed by specific performance standards amplifies the benefits of self-assessment [28–31].

Informed self-assessment describes the “set of processes through which individuals use external and internal data to generate an appraisal of their own abilities” [32]. Learners aware of specific benchmarked standards with access to objective data (i.e., external data) demonstrate improved self-assessment abilities compared to those who rely solely upon their own internal judgments (i.e., internal data) [29–31, 33–35]. Ross et al. proposed a four-stage model to foster learning through informed learner self-assessment that incorporates many of these key elements: (1) involve students in defining the criteria used to judge performance, (2) teach students how to apply the criteria, (3) give students feedback on their performance (informed by objective data) and self-assessments, and (4) help students develop action plans [15].

## Learner self-assessment in debriefing

Critics may question the value of learner self-assessment during debriefing if clinicians struggle with providing accurate self-assessments of their own performance [19]. We argue that such criticism highlights why we should integrate learner self-assessment into debriefing; after all, without having learners self-assess, how will you know how they perceive their own performance? If learners overestimate their own performance, would you not want to know so that you could directly address this misperception? Failure to conduct a learner self-assessment during debriefing places the facilitator at risk for missing out on critical learner misperceptions that may be perpetuated if they are not addressed during the debriefing. Furthermore, the process of learner self-assessment promotes individual and team reflexivity, whereby group members actively “reflect upon … strategies, goals, processes, and outcomes to process key information and adapt accordingly” [14, 36]. Debriefing represents a form of post-action team reflexivity. The plus-delta approach triggers teams to evaluate their performance, which enhances team performance by promoting shared mental models, triggering adaptation, and crystallizing learning [13, 14]. For these reasons, we see a facilitated learner self-assessment as serving a distinctly unique role in debriefing, which emphasizes the importance of being able to conduct a plus-delta during debriefing in a purposeful manner.

Thus, in simulation-based education, debriefing can both engage learners and enhance their capacity for self-assessment in a manner conducive to effective learning. Table 1 provides an overview of how Ross’ four-stage model can foster learning through self-assessment in debriefing [15]. Stage 1 can be achieved during the pre-briefing by having the facilitator review specific performance goals with students and/or introducing a performance checklist for the simulation event [30]. Debriefings offer the optimal venue for addressing stages 2, 3, and 4. To teach learners how to apply performance criteria (i.e., stage 2), facilitators should first conduct a plus-delta with learners and then use language that explicitly connects performance criteria with observed behaviors [15] when closing performance gaps. For example, one strategy would be to view videos of expert modeled performance that demonstrates desired benchmarks [29]. In order to provide feedback on their self-assessments (i.e., stage 3), facilitators should close performance gaps by reviewing performance relative to specific standards (e.g., use of a performance checklist) [30, 31, 33] and generalize discussion to other clinical contexts (i.e., stage 4), both which are tasks central to effective debriefings [2, 12, 37].

**Table 1:** Fostering learning through self-assessment in debriefing using Ross’ four-stage model

Stage	Goal	Activity	Strategies
1. Define the criteria	Clarify criteria used to judge performance	Prebriefing	- Solicit input from learners on potential performance criteria - Review performance criteria—this can be general or specific (e.g., performance checklist or assessment tool)
2. Apply the criteria	Teach learners how to apply criteria in context	Debriefing	- Conduct a plus-delta to obtain a learner self-assessment - Highlight and discuss positive performance - Use language to connect positive behaviors with performance criteria - Review performance checklist or assessment tool relative to performance in simulation - View expert modeled performance (e.g., pre-recorded on video)
3. Provide feedback	Deliver feedback on their performance and reflect on self-assessments	Debriefing	- Identify perception mismatches - Explore and discuss (i.e., focused facilitation) perception mismatches to uncover rationale driving perceptions - Use external data (e.g., video, performance checklists, objective data) to inform feedback - Provide feedback to close performance gaps
4. Develop goals and action plans	Support learners to develop action plans that generalize learning to other contexts	Debriefing	- Discuss how key learning points can be generalized to other clinical contexts - Identify and summarize key learning points/action plan

## The art of the plus-delta

In this section, we introduce four specific considerations when implementing plus-delta, offered in the order of decision-making typically required of a facilitator during a debriefing.

### Assessing the big picture vs. specific performance issues

**Fig. 1:**
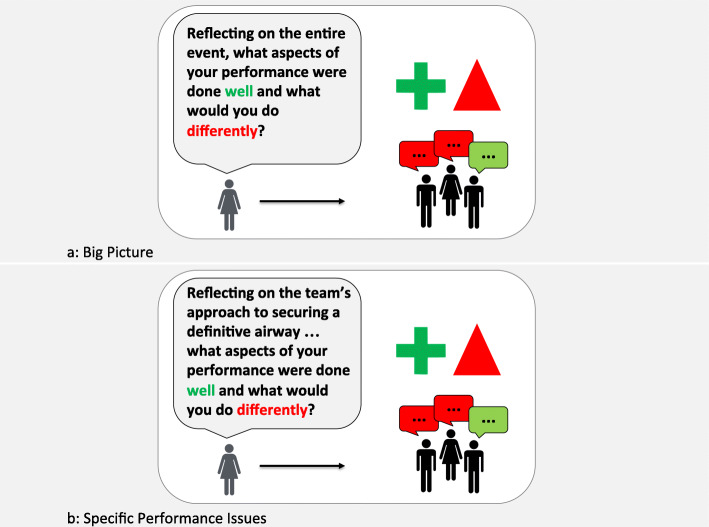
Use of plus-delta for learner self-assessment of: **a.** The big picture or **b.** Specific performance issues

As with other conversational strategies, selective use of plus-delta may be appropriate at various points in discussion depending on the debriefing focus. In a blended method of debriefing, we locate plus-delta during the analysis phase [12, 38]. At the beginning of the analysis phase, facilitators may use a plus-delta to obtain a learner assessment of the “big-picture”, or the entire clinical event (Fig. 1a). In doing this, facilitators identify the learner agenda and recognize perception mismatches early in the analysis phase, which in turn helps prioritize topics for the remainder of the debriefing [18]. Of course, a plus-delta at the beginning of the analysis phase is not always necessary or appropriate. For example, when a rich reactions phase allows identification of numerous topics for discussion, facilitators may forgo a plus-delta and dive directly into focused facilitation. Facilitators should tailor the use of plus-delta to debriefing context (i.e., what has already been discussed) and learner needs.

Alternatively, the plus-delta approach can be used as a tool to explore specific aspects of performance (Fig. 1b). A preview statement preceding the plus-delta question supports the use of the plus-delta approach to unpack specific learner behaviors. For example, the facilitator might say: “I’d like to spend some time discussing the task of defibrillation; and I’d like to get your take before I share mine” as a preview to a plus-delta on how defibrillation was conducted during the simulated cardiac arrest event, which might sound like: “Reflecting on the three instances when you had to defibrillate the patient, can you share what was done really well, and what you would do differently next time?”. Even using plus-delta this purpose, we encourage facilitators to keep in mind the need to identify and further explore perception mismatches as they arise.

### Single- vs. double-barreled questioning

**Fig. 2:**
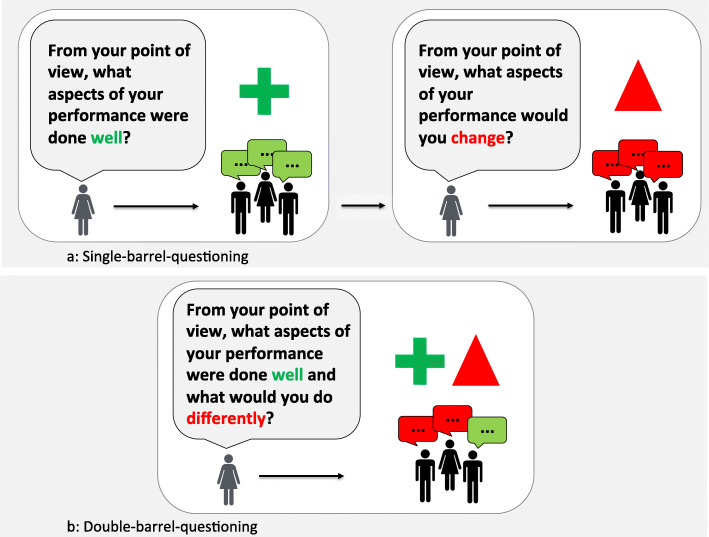
Phrasing of questions in plus-delta for: **a.** Single-barrel questioning or **b.** Double-barrel questioning

We see two main ways of approaching questioning when using plus-delta: single-barreled questioning (i.e., one-part question) and double-barreled questioning (i.e., two-part question). Single-barreled questioning involves asking the “plus” question first (e.g., “What aspects of your performance were done well?”), followed by reflective discussion of each of these points (Fig. 2a). Once completing the discussion of “plus” items, facilitators then pose the “delta” question (e.g., “What aspects of your performance would you change next time?”), followed by facilitated discussion and group reflection. With double-barreled questioning, facilitators asks both the “plus” and “delta” questions back to back (e.g., “What aspects of your performance were done well, and what things would you do differently next time?”), thus leaving it to the learner group to determine what aspects of performance to explore during discussion (Fig. 2b).

We see pros and cons to both approaches. Single-barrel questioning are inherently limiting, conferring more control (of debriefing content) to the facilitator by asking a question with a narrower scope. If, for example, a facilitator is debriefing a team of novice learners who have just performed poorly, they may see value for the learner group to explore positive aspects of their performance first. In this case, posing the “plus” question with the single-barreled approach would serve that purpose. As a downside, this approach exerts more control over the content of discussion may force the conversation in a direction misaligned with learner wishes, particularly when learner performance was sub-optimal (or vice versa). Double-barreled questions allow more freedom of response, placing the onus on learners to identify which aspects of performance, either “plus” or “delta” or both, to highlight during discussion. This approach often uncovers the learner agenda (i.e., the issues that more most important to the learners), which helps facilitators shape future discussion towards learner priorities [18]. Double-barreled questioning risks focusing learner groups entirely on answering only one part of the question (i.e., typically the “delta” question). In situations where learners focus on poor performance, a mentality of “bad is stronger than good” may overtake the debriefing, making it hard to shift gears despite potentially different preferences or perspectives [39]. In some cases, facilitator may never get around to re-asking the “plus” part of the question again, potentially leading to a debriefing that neglects positive aspects of performance.

### Unpacking positive performance

Reflecting on our experiences teaching plus-delta to simulation educators around the world, we have discovered a tendency to focus on discussion of “delta” items at the expense of “plus” items. An inherent assumption drives this behavior, namely that learners derive more value learning from poor performance than good performance [39]. This concept, referred to in psychology literature as negativity bias [40], is especially pronounced when learners feel there is an opportunity to adapt their performance [41], as in simulation. As educators, when we see healthcare teams excel during clinical scenarios, we assume that all team members appreciate that all aspects of the case were managed well and how they were able to collectively achieve those goals. This is a dangerous assumption. When learners do something properly, other learners do not automatically appreciate (a) what was done well, (b) how it was done, (c) and why it was important to be done in that fashion. Failure to explore aspects of positive performance represents missed learning opportunities during debriefing [42].

**Fig. 3:**
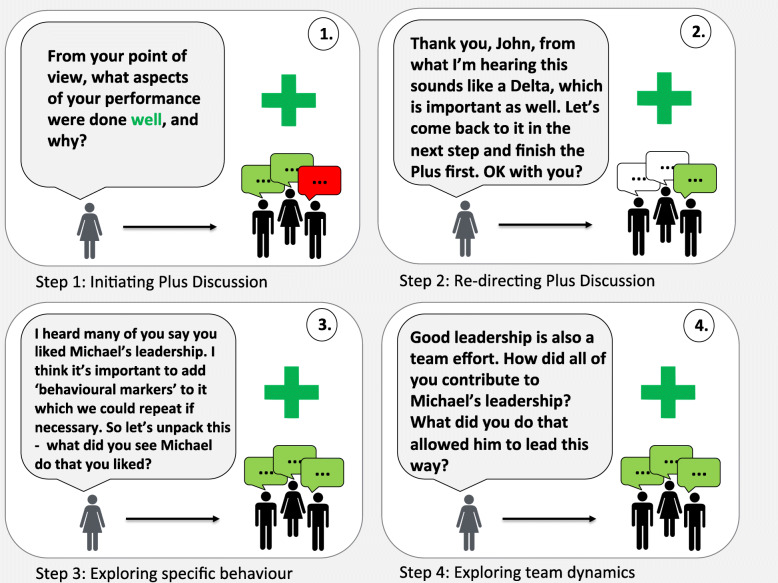
Steps for unpacking positive performance in plus-delta: 1. Initiating plus discussion. 2. Re-directing plus discussion. 3. Exploring specific behavior. 4. Exploring team dynamics

We support Dieckmann et al.’s assertion about the value in unpacking positive performance (i.e., “learning from success”) during debriefings [43] and believe that plus-delta facilitates this activity. Following up the “plus” question with additional probing questions to explore the “what,” “how,” and “why” aspects of performance will deepen learning. For example, in response to the question “What aspects of performance were done well?”, learners may say: “I really thought that Michael did a great job as the team leader – he was awesome!”. To unpack this further, the facilitator could ask: “Tell me more about what you liked about Michael’s leadership”, “What made Michael an effective leader?”, “How did Michael bring you together as a team?”, or “Why was it so important to have a strong leader?” (Fig. 3). Alternatively, a skilled facilitator may further deepen discussion through focused facilitation (e.g., advocacy inquiry [37, 44], circular questions [45]) to explore the underlying rationale for these behaviors [12] (Table 2). All of these approaches encourage learners to reflect deeply on one aspect of the team’s performance, thus ensuring that all learners can carry these positive behaviors through to their next clinical encounter.

**Table 2:** Examples—language to manage perception mismatches in debriefing

Plus-delta question	Preview statement	Focused facilitation
**Single-barreled questions**		***Advocacy inquiry*** [34, 40]
“What were some aspects of your performance that you did well?”	“So, one of the things that I’m hearing is that you guys think that the communication in that scenario went very well. I can understand that, but I’ve also got a slightly different perspective that I would like to share with you.”	“I noticed that there was a lot of communication amongst the team during that scenario, but it seemed to me that several of the key tasks didn’t get completed because they were not specifically given to one team member … I’m concerned that this led to a delay in those key tasks. How did you see it?”
“What would you do differently next time?”	“I’m hearing that you thought that there was too much confusion about what type of shock that you were dealing with in this scenario, and that delayed your ultimate management. I can see your point of view but want to share a slightly different perspective.”	“I saw there was some confusion as to what type of shock you were dealing with as you tried to work it out amongst the team. During this time the patient still got an initial bolus of intravenous fluids, which worries me as that might have been potentially harmful for a patient in cardiogenic shock. Can you share with me your thoughts as you were working through this problem?”
**Double-barreled questions**		***Circular questions*** [41]
“What was easy, and what was challenging for you?	“I’m hearing different perceptions of what was easy and what was challenging. I think this is both normal and important for collaborating as team members. Let’s take a moment and explore these differences further.”	“How do you explain these differences in your perception of challenges?” “In your view, how important is it to agree on these challenges?” “If you were saying ‘OK, I’ll take the lead and I need your help with this’, what do you imagine the other team members would do?”
“From your point of view, what did you do well, and what would you do differently next time?”	“I’m hearing different perception of what went well and what could be done differently. It is very common to see things from one’s own perspective. Highlighting differences is important and why we debrief. Let’s take a moment and explore these differences further.”	“How do you explain these differences in your perception of what went well and what could be improved?” “On which aspects do you agree? What is different in these aspects? On which aspects do you not agree? What’s different here?”

### Managing perception mismatches

One challenge facilitators face is when their assessment of the learner performance differs from the learners’ perception of their own performance. The plus-delta approach captures a small “biopsy” of learner insights. With just one or two questions, facilitators obtain an overview of how learners viewed their own performance, which they can quickly compare with their own personal assessment and/or pre-defined performance measures. In some instances, learners provide a self-assessment that does not agree with the facilitator’s assessment of their performance [19, 22, 23, 25, 46]. This becomes clear when one or more learners categorize behaviors in the “plus” column that the facilitator believes belong in the “delta” column, or vice versa. Here facilitators face a perception mismatch—namely, learners’ believe they have performed well, when in fact they have performed below the standard (or vice versa). Discordant assessments of performance amongst learners thus highlight differences in perception that require further discussion. This is important because people tend to wrongfully assume that others share their perception [47] which prevents them from explicitly discussing them. Reflecting on differences in perceptions allows team members to update team mental models that represent knowledge structures, thus enabling team members to build accurate explanations and expectations of a task [14, 48]. As such, facilitators should prioritize perception mismatches as key learning opportunities during debriefings. Perception mismatches also threaten psychologically safe learning environments. Without the feeling that they can speak their mind, learners may withhold their self-assessment to protect themselves from feared criticism or feel alone with, or even ashamed of, their individual perception [49].

To foster psychologically safe conversations when perception mismatches exist, we encourage facilitators to explicitly introduce the issue with a preview statement: “I’m hearing two slightly different perspectives on the way the team approached airway management. Let’s spend some time reflecting on how and why this unfolded….” A preview statement provides clarity and frames the upcoming portion of discussion for learners. Facilitators may subsequently pose additional probing questions to explore the “what,” “how,” and “why” of their performance, or they may use specific focused facilitation strategies (e.g., advocacy inquiry [37, 44] or circular questions [45) to uncover the rationale driving certain learner behaviors (Table 2). Facilitators help normalize differences in experiences and explicitly appreciate shared self-assessment(s) that seem to stand out or be in the minority. This intervention also helps manage group polarization (i.e., shift towards talking about certain issues while neglecting others) [50]. Through these combined approaches, facilitators gather various perspectives, gain understanding about learners’ rationale for behavior, and work to close gaps in knowledge, skills, or teamwork that contributed to the perception mismatch.

## Discussion

The process of learner self-assessment enables performance improvement, lifelong learning, and most importantly, safe patient care. A genuine connection between the educator and learner fosters learning through the self-assessment process [26]. In debriefing, this connection can be built by ensuring a psychologically safe learning environment through implicit (e.g., body language, eye contact) and explicit strategies (e.g., validation, normalization) [49]. To maximize the benefit of this process, the facilitator should work towards optimizing accurate learner self-assessment.

In describing effective informed self-assessment practices, Epstein et al. highlight that the “power of self-assessment lies in … the integration of high-quality external and internal data” to assess performance [51]. Many debriefings rely heavily (or entirely) upon internal data, or learners’ “self-perception of their performance and emotional state” [31], which relies on personal biases and is often flawed. The incorporation of external data sources (e.g., objective data, performance checklists, and video) into their debriefing conversations can counter biases and misperceptions arising from internal data. Recently published guidelines from the American Heart Association recommend the inclusion of objective CPR data during post-event debriefings, as evidence suggests data-informed debriefing improves provider performance and survival outcomes from cardiac arrest [52]. The impact of using performance checklists as external data sources can be augmented if learners clearly understand these benchmarks, and if learners actively make judgments of their performance using these criteria [30, 53]. The introduction of the performance standards during the pre-briefing, coupled with a plus-delta approach supported by performance checklist review (relative to performance) during the debriefing, would enact this recommendation. Lastly, we see opportunities for the selective use of video as objective, external data to facilitate informed learner self-assessment during debriefing. Video review could potentially clarify misperceptions in performance, or serve to illustrate outstanding performance that meets or exceeds standards [29].

Learner self-assessment, while often fraught with inaccuracies, has clear benefits that can support learning during debriefing. Ross’ four-stage model provides a guiding framework for specific strategies that foster learning through self-assessment in simulation-based education [47]. Facilitators may further master the art of plus-delta by managing perception mismatches, selectively engaging learners in self-assessing performance at either the “big picture” level or for specific performance issues, thoughtfully using single- vs. double-barreled questions, and unpacking positive performance. In providing evidence and strategies for informed learner self-assessment, we hope facilitators will embrace and confidently implement the plus-delta approach to debriefing in a manner that further enhances learning outcomes.

## Data Availability

Not Applicable
